# Antarctic teleosts with and without hemoglobin behaviorally mitigate deleterious effects of acute environmental warming

**DOI:** 10.1371/journal.pone.0252359

**Published:** 2021-11-24

**Authors:** Iskander I. Ismailov, Jordan B. Scharping, Iraida E. Andreeva, Michael J. Friedlander

**Affiliations:** 1 Fralin Biomedical Research Institute at Virginia Tech Carilion, Roanoke, Virginia, United States of America; 2 Virginia Tech Carilion School of Medicine, Roanoke, Virginia, United States of America; 3 Department of Biological Sciences, Virginia Polytechnic Institute and State University, Blacksburg, Virginia, United States of America; Universidade de Lisboa, Faculdade de Ciências, PORTUGAL

## Abstract

Recent studies forecast that many ectothermic animals, especially aquatic stenotherms, may not be able to thrive or even survive predicted climate change. These projections, however, generally do not call much attention to the role of behavior, an essential thermoregulatory mechanism of many ectotherms. Here we characterize species-specific locomotor and respiratory responses to acute ambient warming in two highly stenothermic Antarctic Notothenioid fishes, one of which (*Chaenocephalus aceratus*) lacks hemoglobin and appears to be less tolerant to thermal stress as compared to the other (*Notothenia coriiceps*), which expresses hemoglobin. At the onset of ambient warming, both species perform distinct locomotor maneuvers that appear to include avoidance reactions. In response to unavoidable progressive hyperthermia, fishes demonstrate a range of species-specific maneuvers, all of which appear to provide some mitigation of the deleterious effects of obligatory thermoconformation and to compensate for increasing metabolic demand by enhancing the efficacy of branchial respiration. As temperature continues to rise, *Chaenocephalus aceratus* supplements these behaviors with intensive pectoral fin fanning which may facilitate cutaneous respiration through its scaleless integument, and *Notothenia coriiceps* manifests respiratory-locomotor coupling during repetitive startle-like maneuvers which may further augment gill ventilation. The latter behaviors, found only in *Notothenia coriiceps*, have highly stereotyped appearance resembling Fixed Action Pattern sequences. Altogether, this behavioral flexibility could contribute to the reduction of the detrimental effects of acute thermal stress within a limited thermal range. In an ecologically relevant setting, this may enable efficient thermoregulation of fishes by habitat selection, thus facilitating their resilience in persistent environmental change.

## Introduction

There is increasing concern about the endurance of life forms on Earth in the face of rising environmental temperatures [1, 2]. As part of that awareness, the potential vulnerability of high latitude marine ecosystems, such as the Antarctic shelf, has drawn particular attention [3, 4], as they are likely facing some of the most rapid environmental changes on the planet [5, 6; see, however, 7]. Furthermore, isolated from the rest of the world by the Antarctic Circumpolar Current, inhabitants of this ecosystem have evolved under extremely cold conditions which remained thermally stable for millions of years [8]. In effect, they are thought to have specialized for such a milieu by developing stenothermy, *i*.*e*., traded-off the ability to adjust to even small variations in temperature [9, 10].

Based on the results of experimental studies in a variety of aquatic organisms subjected to acute warming, one perspective on the physiological mechanisms that limit adaptive capacity and thermal tolerance of ectotherms is that cardiac collapse is ultimately responsible for organismal failure at extreme temperatures (see [11] for a Review). Deriving rationale from the relationship between thermal tolerance of fishes and their aerobic metabolic rates [12], these studies attribute this collapse to a mismatch between the aerobic demand of the heart during thermally induced tachycardia and maximal ability to supply oxygen to the heart. However, the ecological relevance of these experiments, which utilize acute sub-lethal warming at miniscule timescales rather than environmentally meaningful thermal limits, remains to be determined. Furthermore, experimental studies in instrumented, often sedated or restrained animals, or in perfused *in vitro* or *in situ* preparations, *i*.*e*., in isolated and/or surgically removed organs, do not take into account behavior, a critical thermoregulatory mechanism for ectotherms [13].

In this study, we examine behavioral responses elicited by ambient warming in two highly stenothermal Antarctic teleosts, *Notothenia coriiceps* (*N*. *coriiceps*, Richardson 1844) and *Chaenocephalus aceratus* (*C*. *aceratus*, Lönnberg 1906), swimming unrestricted in a tank. These fishes belong to the related families *Nototheniidae* and *Channichthyidae*, respectively, but differ in that the blood of *C*. *aceratus* (also called icefish) is devoid of the oxygen transport protein hemoglobin [14, 15]. Consequently, hemoglobinless (Hb-) icefishes supply tissues with oxygen dissolved directly in plasma, with an estimated oxygen-carrying capacity 90% lower than the blood of hemoglobin expressing (Hb+) Antarctic fishes [16]. Large volumes of plasma, big hearts and elaborate vasculature [17] are thought to offset the Hb- state in icefishes, affording their successful habitation in the oxygen-rich Southern Ocean. Yet, compared to their Hb+ relatives, icefishes appear to be less tolerant to acute warming [18], presumably due to inferior capacity to adjust cardiac performance at elevated temperatures [19; see, however, 20]. Hence, it has been predicted that icefishes would be particularly vulnerable to expected global climate change, unless behavioral thermoregulation and/or other physiological plasticity mechanisms alleviate [21] the adverse effects of the challenges associated with it.

Otherwise, while being considered extremely stenothermal, negatively buoyant bottom dwellers *N*. *coriiceps* and *C*. *aceratus* are both in fact eurybathic [22], *i*.*e*., capable of living at a wide range of depths. That is, occasionally found at depths as far down as 500 to 700 meters, respectively, both fishes prefer bathymetric ranges closer to the surface. Namely, *N*. *coriiceps* are most commonly found at depths less than 100 m, while *C*. *aceratus* are typically observed at depths between 100 and 300 m [23], conceivably both performing frequent routine migrations in the water column. In all locations inhabited by both fishes from as far north as South Georgia and Bouvetøya islands (both at ~54°S) to the Antarctic Peninsula (~65.5°S) [23] these depths correspond to a prominent thermo- and oxycline (most significant between 50 and 300 m, S1 Fig), encompassing a range of temperatures from -1.8°C to +2°C and a dissolved oxygen (DO_2_) gradient from ~10 mg·L^-1^ to ~6 mg·L^-1^. Therefore, the fishes must possess certain adaptive capacities that allow them to function equally well in such diverse environments. The physiological mechanisms that provide for these adaptive capacities may be different between Hb + and Hb- fishes, and they may manifest as differences in behavioral thermoregulation. In particular, since locomotion has been considered the main thermoregulatory mechanism of non-sedentary (vertebrate) ectotherms [13], we anticipated locomotor responses to warming to be different in the two species. Supporting this hypothesis, warmed Hb+ *N*. *coriiceps* were noted to become more active, “as if agitated”, while Hb- *C*. *aceratus* remained stationary [24]. We also hypothesized that respiratory adjustments of fishes to progressive aquatic hypoxia and increased metabolic cost at elevated temperatures could be different, in line with differences in oxygen-carrying capacity of blood between the two species, *i*.*e*., Hb+ *N*. *coriiceps* would adjust more readily than Hb- *C*. *aceratus*. Testing these hypotheses constituted the main goal of this study.

Thus, in the course of our investigation, we performed comparative analyses of species-specific repertoires of locomotor and respiratory responses in Hb + and Hb- Antarctic fishes to acute ambient warming. Other outcome measures assessed in this study were serendipitous and included changes in fin movements, kinematics and lateralization of axial body movements, and patterning of maneuvers.

## Materials and methods

### Ethics statement

All animal procedures were approved by the University of Alaska, Fairbanks Institutional Animal Care Committee (570217–9) and Ohio University’s Institutional Animal Care and Use Committee (14-L-004). Experiments were designed around acute warming protocol used previously to study thermal tolerance of Antarctic notothenioids [18]. This protocol constitutes a modification of a classic thermal ramping method introduced in 1944 to study thermal tolerance of desert reptiles by means of so called Critical Thermal Maximum (CT_max_) [25]. The latter was defined as a thermal point at which locomotion of the animal becomes disorganized, leading to the loss of ability to escape from harmful conditions and prompt death. The choice of variables that identify physiologically and ecologically relevant end-points during the state of moribundity (a severely debilitated state close to death of the animal) including that in the CT_max_ method has been a subject of debate [26–29]. Of those, the onset of Loss of Equilibrium (LOE) represents a reasonably humane end-point, because it does not constitute irreversible injury or death of fishes [30]. A comparable end-point, the onset of so called “loss of righting reflex” (LRR), was used in the legacy protocol [18]. However, we settled on the LOE end-point, because detection of the LRR in the legacy protocol involved an intrusive action to disturb the balance of the animal and watching if it was capable of righting itself (as opposed to animal losing equilibrium on its own). Each animal was used in a single behavioral experiment without repetition. Once animals reached endpoint criteria, they were promptly removed from the tank and transferred to other participants of the project for euthanasia and collection of tissues for biochemical assays [31, 32]. The amount of time elapsed from the onset of LOE before euthanasia was always less than three minutes. No animal died before endpoint was reached. A total of ten *N*. *coriiceps* (Richardson 1844) and *C*. *aceratus* (Lönnberg 1906) were used, five of each species. Based on the size of animals, they were all considered adults. There was no discrimination for inclusion of animals in behavioral experiments, with exception of gravid females and individuals with obvious signs of damage and/or distress. Using conventional values of false positive rate α = 0.05 and false negative rate β = 0.20 and the highest possible effect size of 1.0 (like what could be expected for a binary outcome such as emergence/disappearance of behavior that was absent/present before warming), the desired size of each sample of two independent groups was estimated as sixteen [33]. On the other hand, our experimental design of continuous dynamic monitoring (*i*.*e*., in effect, collecting repeated measurements of variables) can increase statistical power for detecting changes in a smaller sample. With all these considerations, however, the actual number of specimens used was based on availability of animals caught in the field, to be effectively shared between all co-investigators in the project.

### Animals

Animals were collected during the austral fall of 2015 (April-June) off the Southwestern shore of Low Island (63°30ʹS, 62°42ʹW) and of Brabant Island in Dallmann Bay near Astrolabe Needle (64°08ʹS, 62°40ʹW). Otter trawls were used to capture both *C*. *aceratus* and *N*. *coriiceps*, and baited benthic traps—for *N*. *coriiceps* only, all deployed from the Antarctic Research and Supply Vessel *Laurence M*. *Gould* (LMG). On board of the LMG, captured animals were held segregated by the species up to 4 days in insulated 900 L tanks (Xactics^TM^, ON, Canada) supplied with running ambient ocean water and superfluous aeration at a rate of 17 L·min^-1^ provided by two submersed glass-bonded silica air diffusers (Sweetwater® model AS5L, Pentair Aquatic Eco-Systems, FL, USA) and two diaphragm air pumps (24 L·min^-1^ at 1 psi output, Sweetwater® model SL24, Pentair Aquatic Eco-Systems, FL, USA) per tank. After transfer to aquaria at the United States Antarctic Program research station, Palmer Station, fishes were kept segregated by the species in 9,000 L tanks flowing fresh sand-filtered ocean water pumped from the Arthur Harbor at ambient temperature (-1.7°C to +1°C) for a minimum period of 72 hours and up to 3 weeks prior to experiments. *N*. *coriiceps* were fed fish muscle blocks once every 2–3 days. Icefish do not feed in captivity [19, 31, 32], and these animals were used within two weeks of capture. A part of the legacy protocol, this was based on prior experience and on the notion that metabolic rate of icefishes is lower than other notothenioids and that they are thought to feed at relatively broad time intervals.

### Temperature ramp experiments

Behavioral experiments were performed in a custom-built 500 L (93 cm (W) x 93 cm (L) x 74 cm (H)) flow-through experimental acrylic tank (S2A Fig) placed in a climate controlled room (with air temperature maintained between +2°C and +4°C) and filled with 300 liters of seawater. All surfaces of the tank were additionally covered with 3.175 mm thick transparent red (#2423) acrylic (Professional Plastics, Inc., CA, USA). The rationale for the latter was based on the finding that Notothenioids lack long-wave sensitive opsin gene [34]. Therefore, used photic conditions mimic light environment corresponding to the austral mid-winter darkness in the natural habitat of the fishes, visually shield them from the experimenters, and should help to minimize stress.

Before the experiment, each specimen was allowed to acclimate overnight in the experimental tank, which was continuously flowing with fresh ocean water (at a rate of 11–15 L·min^-1^) pumped from Arthur Harbor at ambient temperature. This ensured relative consistency of thermal environment of fishes from capture to the beginning of the experiment. Analyses of video recordings made during these periods (lasting as long as 12 hours in some experiments) have demonstrated that such treatments were sufficient to relieve the stress of the fishes in new surroundings, as evidenced by calmness of the animals during the last two-three hours. Thus, behaviors recorded during the last 60 minutes prior to the warming ramp were accepted as baseline. Warming rate of ~3.2°C per hour was achieved by re-circulating tank water through the coil of a custom-made glass heat exchanger (S2B Fig) with a jacket plumbed to a heating bath-pump (AD28R-30, VWR, PA, USA) running in an external closed loop mode. This warming rate was chosen to maintain consistency of experimental conditions with the legacy protocol used to study thermal tolerance in the same species [18]. In this protocol, a 3.6°C·h^-1^ ramp was rationalized as avoiding possible physiological acclimation of fishes to the new temperature regime, as well as delays of changes in core body temperature of animals behind the change in environmental temperature.

During the temperature ramp, before entering the heat exchanger, re-circulated water was aerated at a rate of 17 L·min^-1^ using two submersed glass-bonded silica air diffusers (Sweetwater® model AS5L, Pentair Aquatic Eco-Systems, FL, USA) and a diaphragm air pump (24 L·min^-1^ at 1 psi output, Sweetwater® model SL24, Pentair Aquatic Eco-Systems, FL, USA). Re-circulation was performed through four intakes (one near each corner), to maximize uniformity of temperatures in the tank (see S2A and S2B Fig for detailed view of the experimental tank). In a few preliminary tests without fish in the tank, differences in temperature measured between different areas and depths of the tank were less than 0.2°C.

Digital video images were acquired with Ethovision XT10 tracking software (Noldus Information Technologies, Inc., Netherlands) at a standard frame rate of 30 Hz using a Basler acA 1300-60gm area scan GigE camera (Basler AG, Germany) equipped with a Computar H2Z0414C-MP CCTV lens (CBC Group, NC, USA). The tank contained no gravel or any other substrate, and imaging was performed in a transparency mode (ventral view), in an 86 cm x 86 cm acrylic mirror (Professional Plastics, Inc., CA, USA) placed at a shallow angle (to minimize distortions) 75 cm below the tank. Illumination was achieved with four 30 cm x 30 cm heat- and flicker-free (200W HMI light output) LED flood light panels (model 1x1LS Litepanels, Vitec Group, UK) positioned 50 cm above the tank and waterproofed by lamination from both sides 36% transmission value diffusion filter (# 216, LEE Filters, UK) placed directly over the tank. Water temperature and DO_2_ were recorded synchronously using an Orion Versa Star meter and DO_2_ probe (Thermo Scientific, MA, USA). Warming and video recordings continued until LOE was observed. Depending on the temperature at which LOE occurred in different specimens, duration of experiments with *C*. *aceratus* varied from 4 h 3 min to 4 h 34 min, whereas duration of experiments with *N*. *coriiceps* varied from 4 h 58 min to 5 h 50 min.

Outcome measures assessed in this study include changes in behaviors, such as locomotion, respiratory behaviors, fin movements, axial body movements, their kinematics and lateralization, all analyzed *post hoc* in video recordings acquired in the laboratory experiments at a field station. Of these, locomotion and respiratory opercular movements were considered as “primary” outcome measures.

### Analyses of locomotor activity

Locomotion was analyzed *post hoc* in video recordings acquired in the experiments at a field station using an automated tracking algorithm of EthoVision XT software. Instantaneous velocities (expressed in body lengths per second, BL∙s^-1^) were calculated by the program from instantaneous (*i*.*e*., measured between every two consecutive frames taken 33 ms apart) displacement of the center of the mass of the fish (determined using a proprietary algorithm of the EthoVision software). The average body lengths were 36.91 ± 1.26 cm and 47.36 ± 3.22 cm, for five *N*. *coriiceps* and five *C*. *aceratus*, respectively (mean ± SEM). Elongation ratios of fishes used in our experiments were determined as the ratio of length over width [35], and constituted 8.38 ± 0.24 (mean ± SEM; n = 5) for *C*. *aceratus* and 4.73 ± 0.11 (mean ± SEM; n = 5) for *N*. *coriiceps*.

### Analyses of respiratory behaviors

Opercular movements were analyzed *post hoc* by manual frame-by-frame measurements of fish head width in Adobe Premiere Pro v.5.5.2 (Adobe Inc., CA, USA) projecting video recordings on the screen of a Dell U2410 monitor (1920 x 1200 resolution) at 3.2x magnification. Due to nature of the experiments in a field station, necessitating some of our findings to be serendipitous, no criteria for including/excluding data points during the experiment were established *a priori*. Consequently, as a result of suboptimal contrast in some video recordings, reliable characterization of respiration metrics in *post hoc* analyses was possible only in three specimens of each species.

All metrics were determined unremitted throughout entire experiment, except the moments when high linear or angular velocity of fish movement precluded reliable measurements. Ventilation frequencies (*f*_*V*_) were determined by tallying of opercular movement cycles per minute, and compared for consistency with records of counts taken at 10–15 minute time intervals during actual experiments at Palmer Station. Opercula opening amplitude (OA), a direct proxy metric of ventilatory stroke volume, was measured as the difference in width of fish head with opercula maximally open and maximally closed, using an Apollo VCG7070 transparency film (Acco Brands, IL, USA) with a 1 mm grid. Accuracy of head width change measurements was arbitrarily set at 0.5 mm. Opercula opening time (OT), an inverse proxy metric of branchial pump suction, was calculated from the number of frames taken during transition of opercula from closed to maximally open state. Opercula opening velocity (OV) calculated as OV = OA/OT. This ratio is considered as “unidimensional” proxy metric of branchial pump velocity to characterize the efficacy of ventilation.

Numbers and durations of surfacing events were manually tallied *post hoc* on the frame-by-frame basis in Adobe Premiere Pro.

### Kinematics of startle-like behaviors

Spontaneous startle-like behaviors were analyzed *post hoc* manually in Adobe Premiere Pro, on a frame-by-frame basis, using conventions comparable to those described above for analyses of respiratory behaviors and Apollo VCG7070 transparency film (without a grid) placed directly on a Dell U2410 monitor projecting video recordings. Angular velocities were determined by measuring with a transparent protractor angles between vectors drawn from the center of mass (vertex of the angle, determined using a proprietary algorithm of the EthoVision software) of the fishes to the snout at appropriate temporal resolution. For fast S-bend startle-like maneuvers of *N*. *coriiceps*, a 33 ms^-1^ frame rate may result in underestimation of angular velocity.

Analyses of axial movements during thermally induced startle-like responses were done using manual sketches of the body shapes of the fishes made on transparency film on a frame-by-frame basis. For presentation purposes, ventral outlines of the turning fish were made over scanned and imported into CorelDraw (Corel Corporation, ON, Canada) images.

### Lateralization of C-bend maneuvers in response to ambient warming

For laterality analyses, quantities and directions of C-bend maneuvers were tallied for every 0.5°C increment of the temperature ramp in each of five experiments with Hb+ and Hb- fishes. Since tank walls can affect lateralization [36], turns that occurred near the walls were not tallied. An arbitrary cut-off distance of approximately one half of body length of a specimen was used, as it was assumed to provide sufficient freedom of choice of directionality.

Relative Lateralization indices (LR, %, [37]) were calculated using following equation:

$$LR\;=\;\frac{\left(Number\;of\;Turns\;to\;the\;Right\;-\;Number\;of\;Turns\;to\;the\;Left\right)}{\left(Number\;of\;Turns\;to\;the\;Right+\;Number\;of\;Turns\;to\;the\;Left\right)}\;\times 100$$

We note, however, that unlike in detour paradigm of Bisazza et al. [37] analyzing single instance of behavior of each fish in multiple experimental trials, we analyzed multiple maneuvers of each specimen in a single continuous warming experiment within the range of temperatures in which the fish demonstrated spontaneous, repetitive startle-like behaviors. Mean LR (± SEM) was used to assess turning preference (i.e. bias in left or right turns) for 5 specimens of each species. The LR index at the level of individuals allowed to classify specimens between the extreme values of “100” (when fish turned right in all cases recorded within temperature interval corresponding to 5% T_LOE_) and “-100” (when fish turned left on all cases recorded within temperature interval corresponding to 5% T_LOE_). A mean LR near zero indicates that a given species is neither left nor right biased in its tendency to turn in a given temperature range.

### Analyses of pectoral fin movements

Duration of fanning bouts, fanning frequency (as a number of pectoral fin beats per second within a bout) and duration of pectoral fin splay were manually tallied *post hoc* on the frame-by-frame basis in Adobe Premiere Pro.

### Quantification, presentation of grouped data, and statistical analyses

Microsoft Office Excel 2010 and 2016 were used for all quantitative analyses and statistical tests. No randomization was used to allocate animals to “control” and “treatment” groups, since each experimental animal had baseline behavior as its own internal control. Likewise, no controls for potential confounders (such as the order of treatments and measurements) or “blinding” of the experimenter(s) were necessary.

For analyses and presentation of grouped data for various behaviors as a function of temperature, data obtained in individual experiments within the species were aligned against normalized range of the ramp (% T_LOE_, bottom axes in Figs 3 through 7, with 0 and 100% corresponding to initial temperature and T_LOE_, respectively, in a given experiment). Such treatment of the data was necessary to account for the differences in initial temperatures and in the LOE onset temperatures between experiments. Absolute ranges of temperature ramps in °C (top axes in Figures) are depicted as red plots above the traces, with red triangle symbols and horizontal error bars representing mean and SEM of the temperatures at the start of the ramp and at LOE averaged between respective numbers of experiments with each species.

Statistical significance of laterality bias was estimated by comparing lateralization indices at each 5% LOE temperature interval to a theoretical zero (random choice, 0% laterality) using one sample *t*-tests [38]. Two sample unequal variance two-tailed *t-*tests were used to determine statistical significance of changes in the temperatures of the onset of the LOE. Analyses of correlation of changes in ventilation metrics were performed using Data Analyses Toolpack routine of the MS Excel.

## Results

### Locomotor responses to warming have alternating patterns

**Fig 1:**
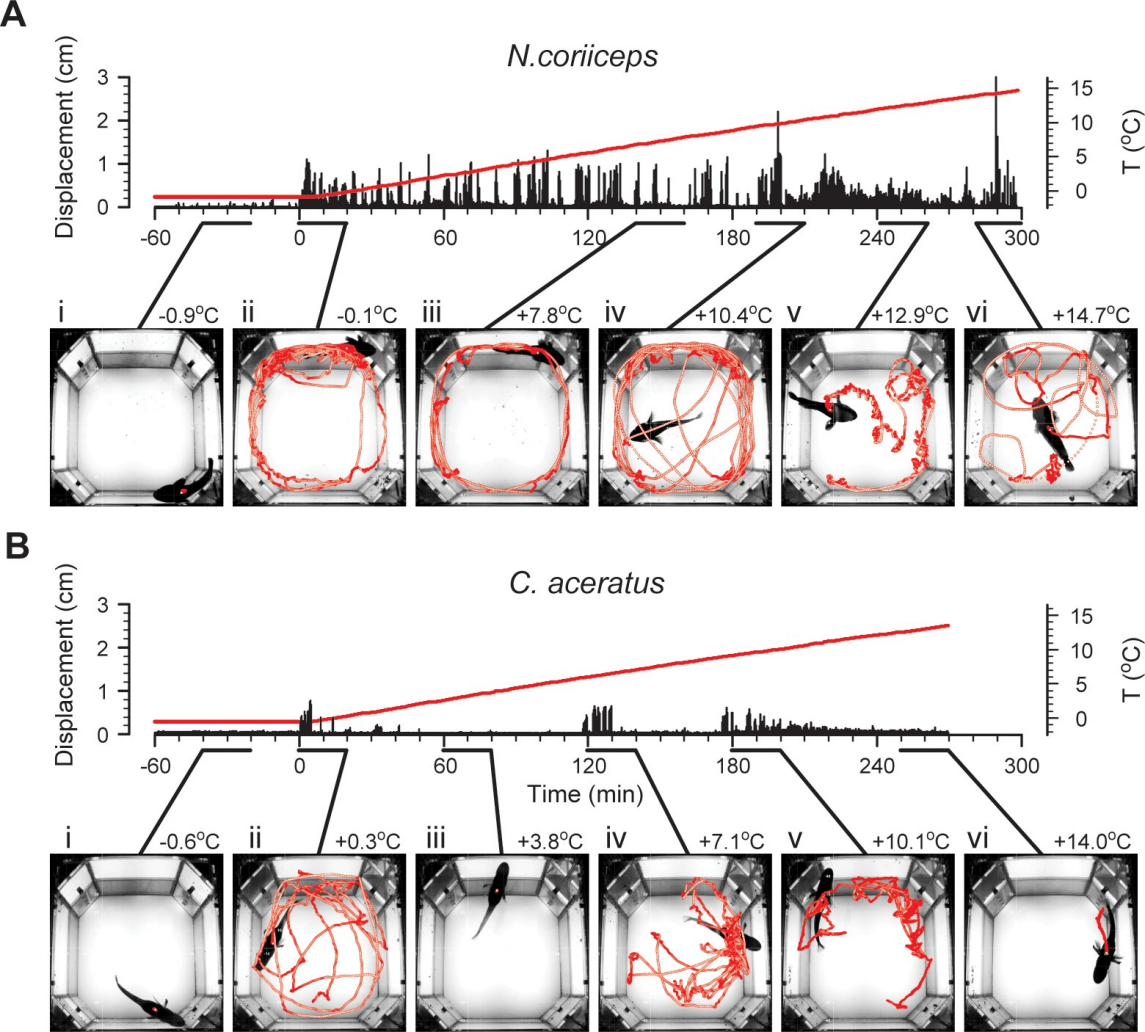
Representative locomotor responses of Hb+ *N*. *coriiceps* and Hb- *C*. *aceratus* to warming. **(A)** and **(B)** represent time plots of absolute instantaneous displacement (left axes) of a single specimen of *N*. *coriiceps* and of a single specimen of *C*. *aceratus*, respectively. Time “0” corresponds to the beginning of the temperature ramp; negative values correspond to the final sixty minutes of an overnight acclimation in the experimental tank. Red lines represent absolute temperature (right axes) of water, with the maximum corresponding to the LOE. Insets below time plots depict locomotion trajectories for 20 minute time periods at select temperatures (maximal temperatures during these episodes are shown next to the insets). (i) Baseline station-holding. (ii) Early responses to initial rise in temperature. (iii) First period of reduced motility. (iv) Intense locomotion and startle-like behaviors. (v) Second period of reduced motility with multiple startle-like behaviors. (vi) Onset of LOE.

Representative locomotor behaviors of *C*. *aceratus* and *N*. *coriiceps* elicited by acute warming, with marked transient bouts of increased locomotion of both species, interspersed with periods of reduced motility are shown in Fig 1. Individual motility varies substantially within each species before and during warming, with Hb+ fish displaying more agility at all times. However, the alternating pattern of thermally induced changes in locomotion persists in all individuals with some species-specific trends in the onset, duration and velocity (S3 Fig). Also seen in S3 Fig, temperatures of the onset of LOE vary between animals and constitute +13.8 ± 0.4°C in *C*. *aceratus* and +15.8 ± 0.4°C in *N*. *coriiceps* (Mean ± SEM, n = 5 for either species; for numerical data of T_LOE_ values in individual experiments, see S1 Table), statistically different between the species (*t*_*crit*_ = 2.31; df = 8; *p* = 0.009; two-sample unequal variance two-tailed *t-*test).

The first locomotor responses to warming occur in all animals of both species with temperature elevations as little as 0.1°C. In striking contrast to sustained sedentary baseline behavior (Fig 1A (i) and 1B (i)), these maneuvers manifest as yawing along the walls of the tank (Fig 1A (ii) and 1B (ii)). Instantaneous velocities up to 0.8 body lengths per second (BL·s^-1^, see Materials and Methods for numerical BL data) in Hb+ and up to 0.4 BL·s^-1^ in Hb- fish are achieved predominately in the labriform swimming mode, with fore-aft rowing strokes of pectorals providing the thrust. Tail and trunk muscles are recruited only for occasional surfacings and short bouts of subcarangiform swimming, mostly evident in Hb+ fish.

Unable to perform thermoregulation by habitat selection in the tank, fishes respond to continued increase in temperature by reduction of motility (Fig 1A (iii) and 1B (iii)). Although variable at the individual level, reduction of motility has marked commonalities within each species. Namely, as the temperature rises 1–1.5°C above initial, Hb- fish assume essentially motionless station-holding and maintain it for tens of minutes. In contrast to that, Hb+ fish generally continue to yaw along the tank walls, but gradually decrease the duration of swimming bouts. By +6°C, locomotion of Hb+ fish subsides, with station-holding episodes lasting up to ten minutes, interleaved occasionally with brief bouts of labriform swimming at velocities not exceeding 0.8 BL·s^-1^.

### Continued warming triggers startle-like responses

Conspicuous episodes of increased locomotion also occur between +5°C and +10°C in Hb- *C*. *aceratus*, and between +8.5°C and +12°C in Hb+ *N*. *coriiceps*, and manifest as intense swimming along the walls, accompanied by multiple surfacing events, particularly in Hb+ fish. Fishes use mainly the labriform swimming mode, achieving linear velocities up to ~1 BL·s^-1^ in Hb+ and ~0.6 BL·s^-1^ in Hb- animals (S3 Fig). In addition, characteristic only for *N*. *coriiceps*, they demonstrate several fast crossings of the tank, through its middle (Fig 1A (iv)), using the subcarangiform propulsion with velocities reaching 2–3 BL·s^-1^ (S3A Fig).

**Fig 2:**
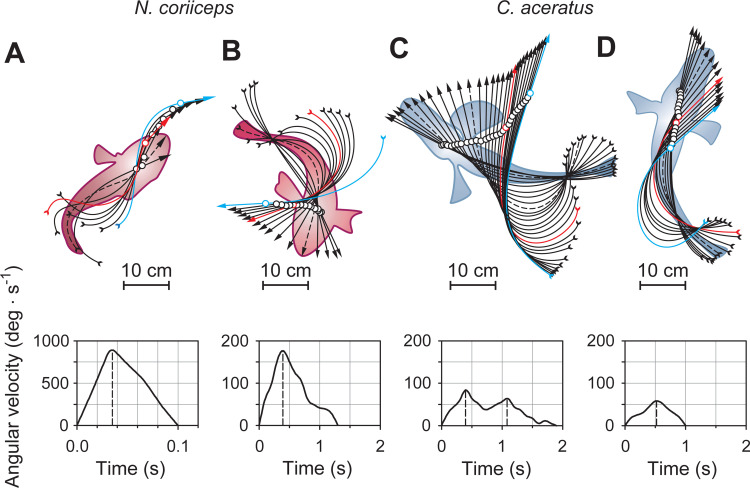
Repertoire of thermally induced startle-like maneuvers of Hb+ *N*. *coriiceps* and Hb- *C*. *aceratus*. **(A)** Fast S-bend turn of *N*. *coriiceps* (33 ms intervals) at +11.1°C. **(B)** Intermediate velocity single-stage C-bend turn of *N*. *coriiceps* (100 ms intervals) at +12.4°C. **(C)** Slow multi-stage C-bend turn of *C aceratus* (100 ms intervals) at +8.5°C. **(D)** Withdrawal-like maneuver of *C*. *aceratus* (100 ms intervals) at +11.5°C. Curved arrows and circles represent midlines and centers of mass of the fishes, respectively. Silhouettes with dashed midlines represent body-shapes and positions of fishes at maximal angular velocities during the turn stage of the maneuvers (dashed vertical lines in the time plots of angular velocity). Red midlines indicate completion of the first stage of the maneuver (namely, the S- or C-bend proper) and transition to the second stage, swimming or gliding without change in direction (shown as blue midlines).

**Fig 3:**
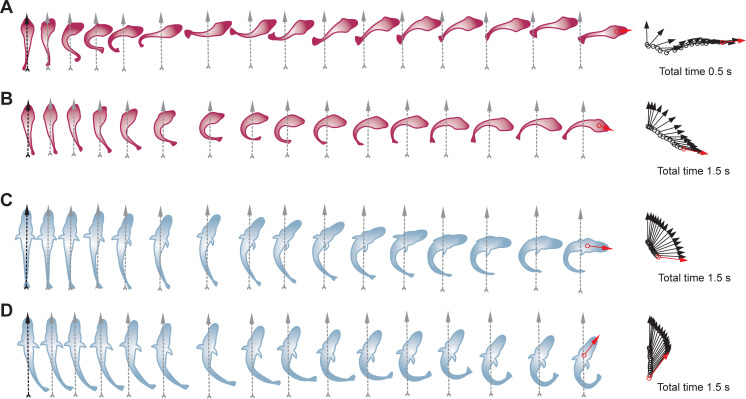
Axial movements during temperature induced startle-like responses in Hb+ *N*. *coriiceps* and Hb- *C*. *aceratus*. **(A)** *N*. *coriiceps* during an S-bend maneuver shown at 33 ms intervals (at +11.05°C). **(B)** *N*. *coriiceps* during a C-bend turn shown at 100 ms intervals (at +12.4°C). **(C)** *C*. *aceratus* during a C-bend turn shown at 100 ms intervals (at +8.5°C). **(D)** *C*. *aceratus* during a withdrawal-like maneuver shown at 100 ms intervals (at +11.5°C). In all panels, ventral views (pectoral fins not shown) are depicted. Gray vertical arrows indicate the position of the midline at the beginning of turning maneuvers. Arrow diagrams on the right depict of the head orientation during turns, at time intervals indicated above. Each arrow connects the position of the fish nose with the center of mass. Red arrows indicate final direction of locomotion.

Notably, most of these maneuvers involve distinctive patterns of body bending and subsequent turning. Namely, high-velocity swimming laps of Hb+ fish begin with a rotation of the head coincident with a typical contralateral tail bending, thus forming an S-shape (Figs 2A and 3A). These highly dynamic S-bends are followed by very fast turns with angular velocities (V_a_) of ~1,000 degrees per second (deg·s^-1^). These maneuvers, however, are scarce, with no more than five of them occurring in each animal. Turns of another type, recurrent in both species, begin with rotation of the head followed by ipsilateral bending of the tail, thus forming a C-shape (Figs 2B, 2C, 3B and 3C). Such C-bend turns of *N*. *coriiceps* occur in a single stage, reaching maximal V_a_ of ~250 deg·s^-1^ (Fig 2B). In contrast, multiple peaks of V_a_ below 100 deg·s^-1^ are evident during C-bend turns of *C*. *aceratus* (Fig 2C). In both species, these maneuvers are followed by either a short labriform swimming bout, or an unpowered glide of variable duration.

While these features are reminiscent of S- and C-starts in escape and startle behaviors reported in other fishes in response to external stimuli [39–41], 30 frames·s^-1^ sampling rate is suboptimal and may limit sufficient temporal resolution of body shapes. However, the midline and the silhouette of the fish in Fig 2A have a discernible S-shape appearance at 33 ms, *i*.*e*., at maximal angular velocity resolved during the turn stage. In addition, translation of the center of the mass of *N*. *coriiceps* during maneuvers depicted in Fig 2A and 2B are consistent with early definitions of S-starts being characterized by displacement in line with the original body axis, and C-starts featuring large angles of turn [39, 40, 42]. Nonetheless, since the fish are not startled by an obvious stimulus, we term these thermally-induced maneuvers “startle-like”, albeit differentiating initial S- and C-bends.

Unique to Hb- species, another type of thermally induced reactive behavior features only slight rotation of the head with V_a_ below 50 deg·s^-1^, followed by retraction of the body (Figs 2D and 3D). Observed in all five specimens of *C*. *aceratus*, it has the appearance of a slow backward movement of variable duration, and resembles “withdrawal” or “head retraction”, a startle response described in some other sedentary bottom-dwelling fishes with elongated bodies [35, 43, 44]. While *C*. *aceratus* are indeed characterized by relatively high elongation ratios (see Materials and Methods for quantification and numerical data) and bottom-dwelling lifestyle, these thermally induced maneuvers are much slower (~1 second in duration, Fig 2D) than canonical withdrawals (which are over in 100–200 ms, [35, 43, 45]). Therefore, we term this behavior “withdrawal-like”.

**Fig 4:**
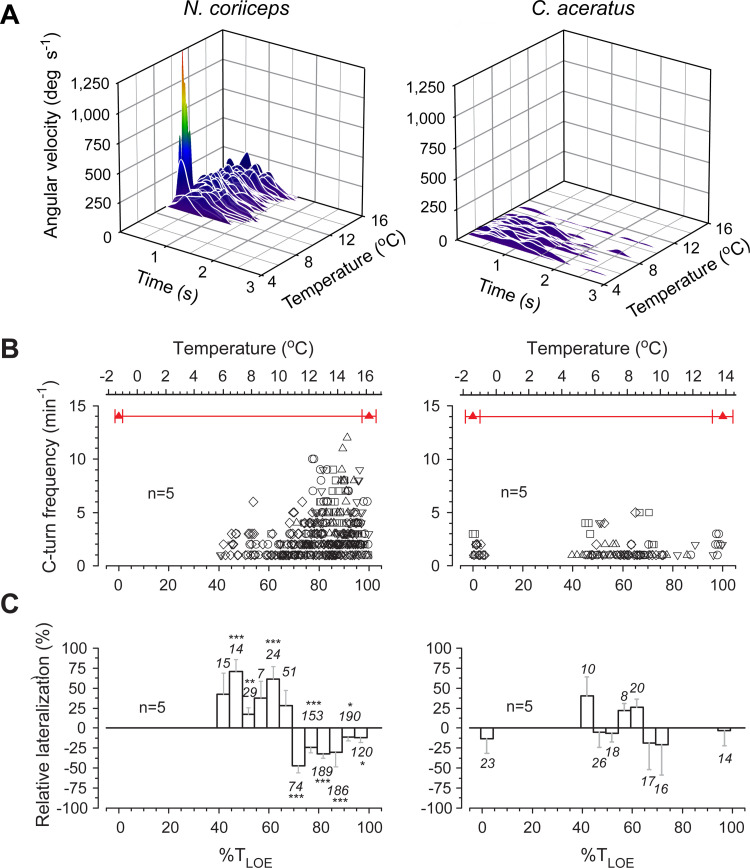
Startle-like behaviors of Hb+ N. coriiceps and Hb- C. aceratus as a function of temperature. **(A)** Time plots of angular velocity (V_a_) during startle-like maneuvers at respective temperatures in a single specimen of *N*. *coriiceps* and *C*. *aceratus*. Rainbow color code denotes the continuum of angular velocities from low (violet) to high (red) during fast S-bend turns (V_a_>500 deg·s^-1^, 9 events) and intermediate velocity single-stage C-bend turns in *N*. *coriiceps* (V_a_<250 deg·s^-1^, 137 events), and during slow multi-stage C-bend turns (V_a_<100 deg·s^-1^, 25 events) in *C*. *aceratus*. **(B)** Data points represent number of C-bend turns during each consecutive time interval one minute in duration in five specimens (depicted as different symbols) of each species. **(C)** Relative lateralization (mean and SEM) of C-bend turns in 5% of T_LOE_ increments averaged between five specimens of each species. For conventions of quantification and presentation of grouped data as a function of temperature, see Materials and Methods. Numerals next to bars indicate numbers of events within each temperature increment in five animals. Asterisks denote statistical significance of laterality bias (*p* values between 0.024 and 0.032 - *; *p* = 0.0012 - **; *p*<0.001 - ***) at each temperature interval compared to a random choice (0% laterality) using one sample *t*-tests.

The repertoire and incidence of these startle-like behaviors of Hb+ and Hb- fishes demonstrate marked differences in thermal dependence (Fig 4). While exact temperatures of the onset of S-bend maneuvers of *N*. *coriiceps* vary between individual specimens, in each animal they occur within a relatively narrow interval of thermal change of ~1.5°C anywhere between +9.5 and +12°C. The intense swimming subsides after that in all Hb+ fish, followed by another period of reduced locomotion. However, they begin performing spontaneous C-bend maneuvers, progressively increasing in incidence, and reaching up to 10 turns per minute between +12°C and +14°C (Fig 4B).

C-bend turns of *C*. *aceratus*, in contrast, are sporadic, and their frequency does not appear to change with temperature (Fig 4B). In this species, scarce withdrawal-like maneuvers constitute the majority of motoric behaviors between +9°C and +12°C, occasionally interrupting extended episodes of station-holding. Nonetheless, startle-like behaviors persist in both fishes until the onset of LOE, when a short bout of erratic locomotion and surfacing occurs in all Hb+ and some Hb- animals (Fig 1A (vi) and 1B (vi)).

Notably, below +11°C, startle-like C-bend turns of all *N*. *coriiceps* display a marked rightward preference (Fig 4C, left graph). Above this temperature, this bias reverses, and the newly established leftward preference persists in subsequent C-bend turns of all five Hb+ fish. With further warming, however, this lateralization gradually decreases and eventually disappears, when turns become scarce near the T_LOE_. Both the initial and reversed biases of turns of *N*. *coriiceps* are statistically significant (for *p*-values for each 5% LOE interval, see Fig 4C, left graph), except when the behavior just starts to manifest at 40% LOE, and at the point of right-left shift near 65% LOE. In contrast, none of five Hb- fish examined demonstrate any apparent bias of thermally induced C-bend maneuvers (Fig 4C, right graph).

### Coping with thermal stress involves ventilatory adjustments

**Fig 5:**
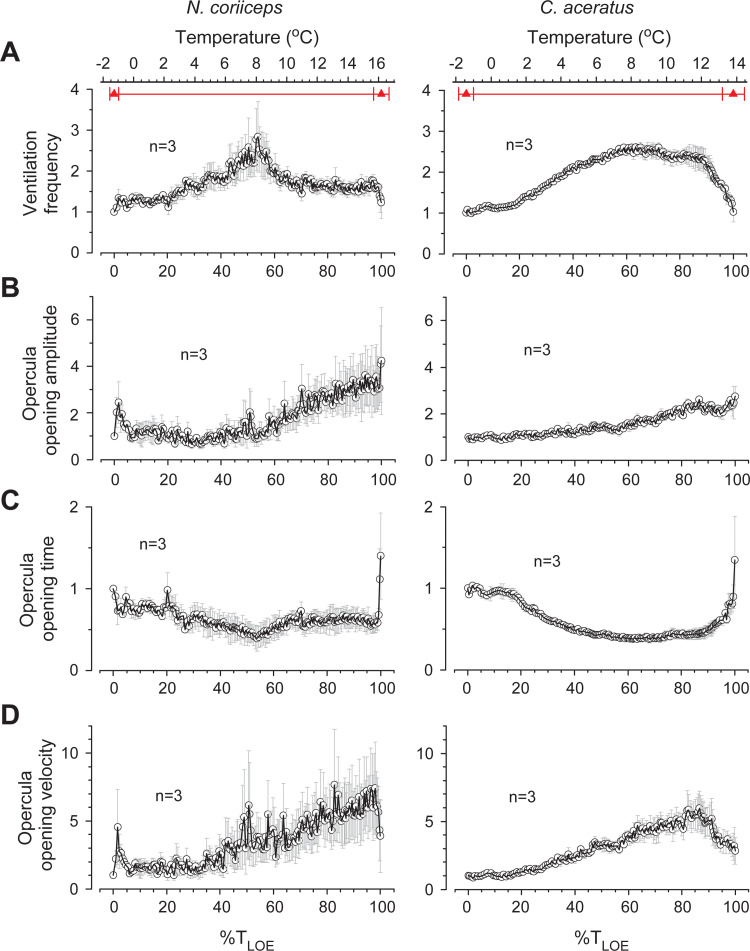
Thermally induced ventilatory responses in Hb+ *N*. *coriiceps* and Hb- *C*. *aceratus*. **(A)** Ventilation frequencies (*f*_*v*_). **(B)** Opercula opening amplitudes (OA). **(C)** Opercula opening times (OT). **(D)** Opercula opening velocity (OV). Data points and error bars in all plots represent means and SEM of each metric normalized to the initial value for each animal, and averaged for three specimens of each species. For conventions of quantification and presentation of grouped data as a function of temperature, see Materials and Methods.

In response to progressive aquatic hypoxia (S4 Fig) that is accompanied by escalating metabolic load associated with rising temperatures, fishes adjust their ventilation, as quantified in *post hoc* analyses of opercular movements in video recordings. Metrics of ventilation in three specimens of each species are shown in S5 Fig and in S2 Table. At all temperatures, absolute ventilation rates of *N*. *coriiceps* are nearly two-fold higher than those of *C*. *aceratus* (S5A Fig). Nonetheless, there are certain similarities in the overall dynamics of changes in ventilation both within and between the species. Namely, after a small transient rise in ventilation frequency (*f*_*V*_) at the onset of warming ramp, once the temperature rises more than 2°C above initial, a steady increase in *f*_*V*_ becomes evident in all animals (Fig 5A and S5A Fig). This hyperventilation persists even during episodes of relative quiescence, which suggests that it is not a consequence of locomotor effort. In both species, *f*_*V*_ reach their maxima between +8 and +8.5°C, rising 2.5±0.9 times in *N*. *coriiceps* and 2.61±0.19 times in *C*. *aceratus* (normalized to the initial for each specimen; the increase is not statistically different between the species). In addition, at the temperatures between +6 and +12°C, near the maximum of *f*_*V*_, both fishes exhibit another well-known type of respiratory behavior, aquatic surface respiration (S6 Fig). Ventilatory responses to further warming diverge between Hb+ and Hb- fishes, but there is a correlation of the overall changes in *f*_*V*_ within the species, stronger in *C*. *aceratus* (*r* ranging from 0.88 to 0.96) than in *N*. *coriiceps* (*r* ranging from 0.40 to 0.71). Namely, after reaching the maximum, *C*. *aceratus* continue to hyperventilate until the 1–1.5°C prior to LOE, whereas *f*_*V*_ of *N*. *coriiceps* declines precipitously within a rather narrow interval of temperature rise (~2°C) to a plateau at a lower, yet still relatively elevated, level.

Two other measures of opercular movements are the opening amplitude (OA) and the opening time (OT). With rising temperatures, OA increases progressively in both species, persistent till LOE (Fig 5B). Hb- fish, however, recruit this adjustment at the onset of hyperventilation, whereas Hb+ fish employ it when *f*_*V*_ approaches its peak. Thermally induced changes of OT, however, are biphasic, and nearly mirror the inverse of changes in *f*_*V*_ in both species (Fig 5C), reaching their minima nearly coincident with the maximal *f*_*V*_ between +8 and +8.5°C. With further warming, *C*. *aceratus* maintain fast opercular openings (short OT) until the last 1–1.5°C prior to LOE, whereas in *N*. *coriiceps* they become slower (longer OT).

Furthermore, during *f*_*V*_ plateaus, in both species, OT remains essentially unchanged, whereas OA steadily rises. This translates into continued growth of the ratio of these two measures of opercular movements, *i*.*e*., opercula opening velocity (OV) (Fig 5D), which keeps increasing while *f*_*V*_ remains constant. When approaching LOE, at the temperatures above +15.5°C in *N*. *coriiceps*, and +11.5°C in *C*. *aceratus*, both *f*_*V*_ and OV decline in both species followed by respiratory collapse.

### Continuous acute thermal stress triggers fanning and fin splay

Two other distinctive behaviors manifest in both species at the temperatures above +8°C. Both of them involve movements of pectoral fins with marked thermal dependences and species-specific differences in appearance.

**Fig 6:**
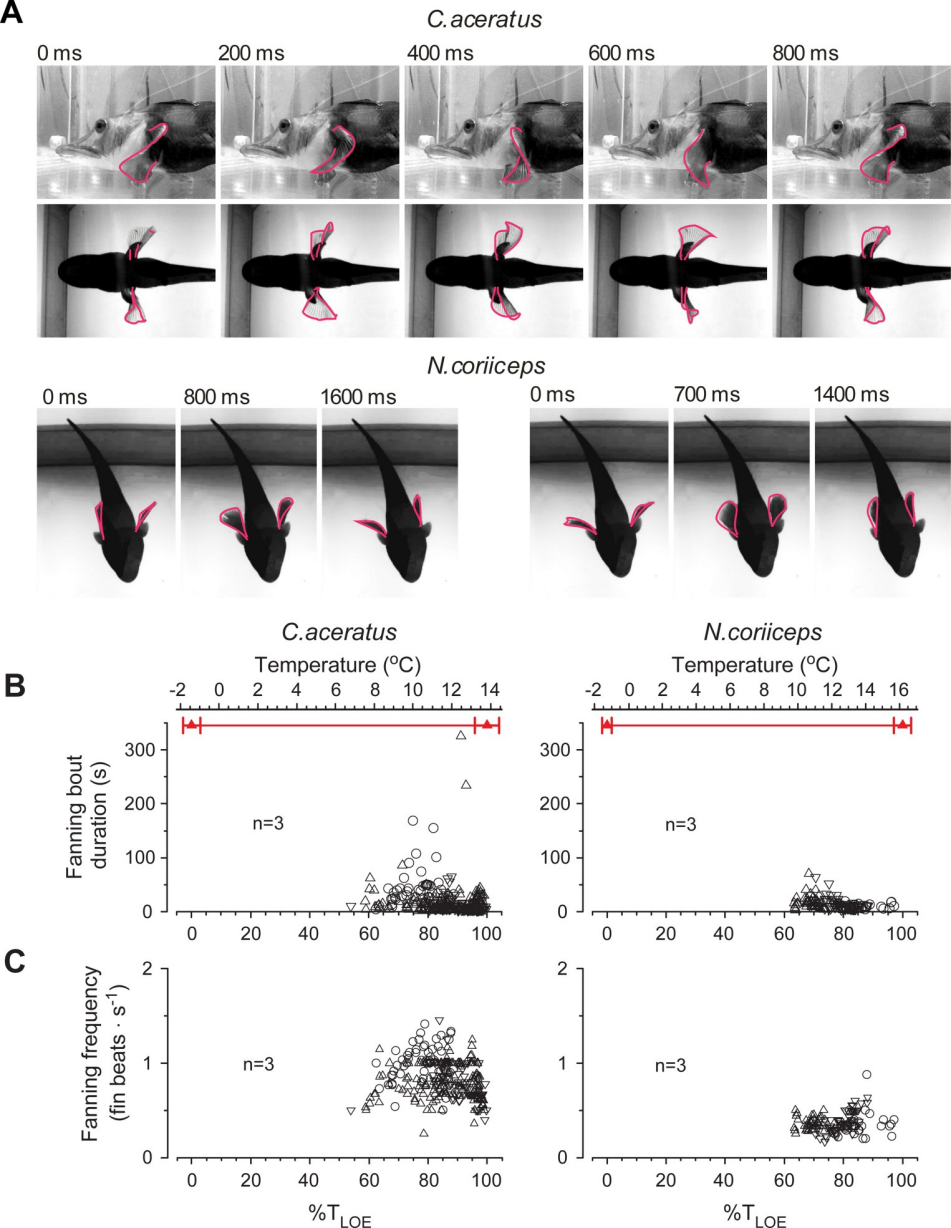
Acute warming elicits pectoral fin fanning in Hb- *C*. *aceratus* and Hb+ *N*. *coriiceps*. **(A)** Top panel depicts lateral (upper images) and ventral (lower images) view of one complete cycle (800 ms in duration, every 6th frame shown) of undulatory fanning, typical for *C*. *aceratus*. Bottom panel depicts ventral view of one cycle of “sway” (fins adducted and abducted in a counterphase) fanning 1.6 s in duration (left set of images, every 24th frame is shown) and one cycle of “sweep” (fins adducted and abducted in a synphase) fanning 1.4 seconds in duration (right set of images, every 21st frame is shown), characteristic for *N*. *coriiceps*. **(B)** Temperature plots of duration of fanning bouts (episodes of uninterrupted continuous fanning at near constant frequency). **(C)** Temperature plots of fanning frequency (number of fin beats per second within a bout). For conventions of quantification and presentation of grouped data as a function of temperature, see Materials and Methods. Different symbols in **(B)** and **(C)** represent three specimens of each species.

One behavior manifests as a cyclical fin movement in nearly stationary fishes, with no obvious relevance to locomotion, comparable to those reported in a variety of fishes during egg-guarding [46] and termed “fanning”. In Hb- fish, it consists of anteroposterior undulations of large and flexible fan-shaped appendages, extended from the trunk (Fig 6A, top panel, and S1 and S2 Movies). These undulations occur in bouts lasting from tens to hundreds of seconds (Fig 6B), and have a mean frequency of ~1Hz (Fig 6C). Movements of less flexible pectorals of Hb+ fish present as co-mingled “sway” and “sweep” motions (Fig 6A, bottom panel, and S3 Movie). They occur in short bouts (Fig 6B), and are more than twice as slow as undulations in *C*. *aceratus* (Fig 6C).

**Fig 7:**
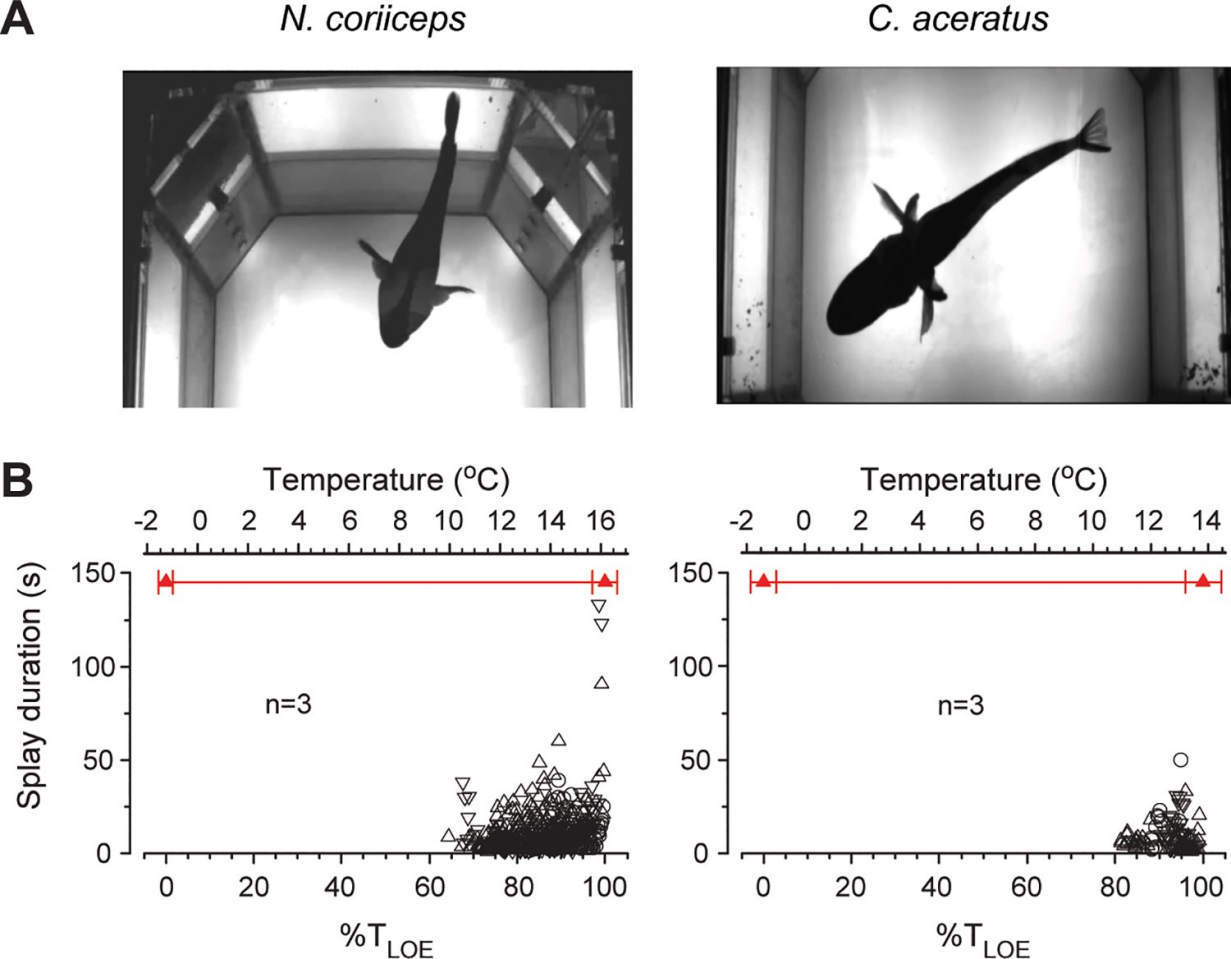
Fin splay behavior at elevated temperatures in Hb+ *N*. *coriiceps* and Hb- *C*. *aceratus*. **(A)** Representative still images of splays in N. coriiceps and C. aceratus. **(B)** Temperature plots of fin splay episode duration. Different symbols represent three specimens of each species. For conventions of quantification and presentation of grouped data as a function of temperature, see Materials and Methods.

Another behavior of fishes manifests in spreading pectoral fins laterally, nearly perpendicular to the trunk, and maintaining this position for a period of time (Fig 7A). To the best of our knowledge, no comparable maneuver has ever been reported before, and we term it “splay” to depict the spreading of appendages. Numerous splays of *N*. *coriiceps* are evident within relatively wide thermal range between +10°C and +16°C, increasing ~10 fold in occurrence by +13°C and up to 5-fold in duration by +16°C (Fig 7B, left panel). Sporadic splays of *C*. *aceratus*, on the other hand, manifest between +9°C and +13°C, increasing ~4 fold in occurrence and up to 3-fold in duration (Fig 7B, right panel).

### Patterned respiratory-locomotor coupling of *N*. *coriiceps*

Numerous fanning and splays of *N*. *coriiceps* are interspersed with C-bend turns, with certain stereotypy in the sequence of maneuvers (S4 Movie), thus having features of “Fixed Action Pattern” (FAP) behavior [47]. Namely, this behavior manifests as copious repetitive Splay-Turn-Glide triplet sequences as often as five-six (up to ten near +14°C) times per minute (Fig 4B). The sequence of the three components remains invariant with increasing temperature, whereas the frequency of triplet occurrence, as well as duration of individual components appear to vary.

**Fig 8:**
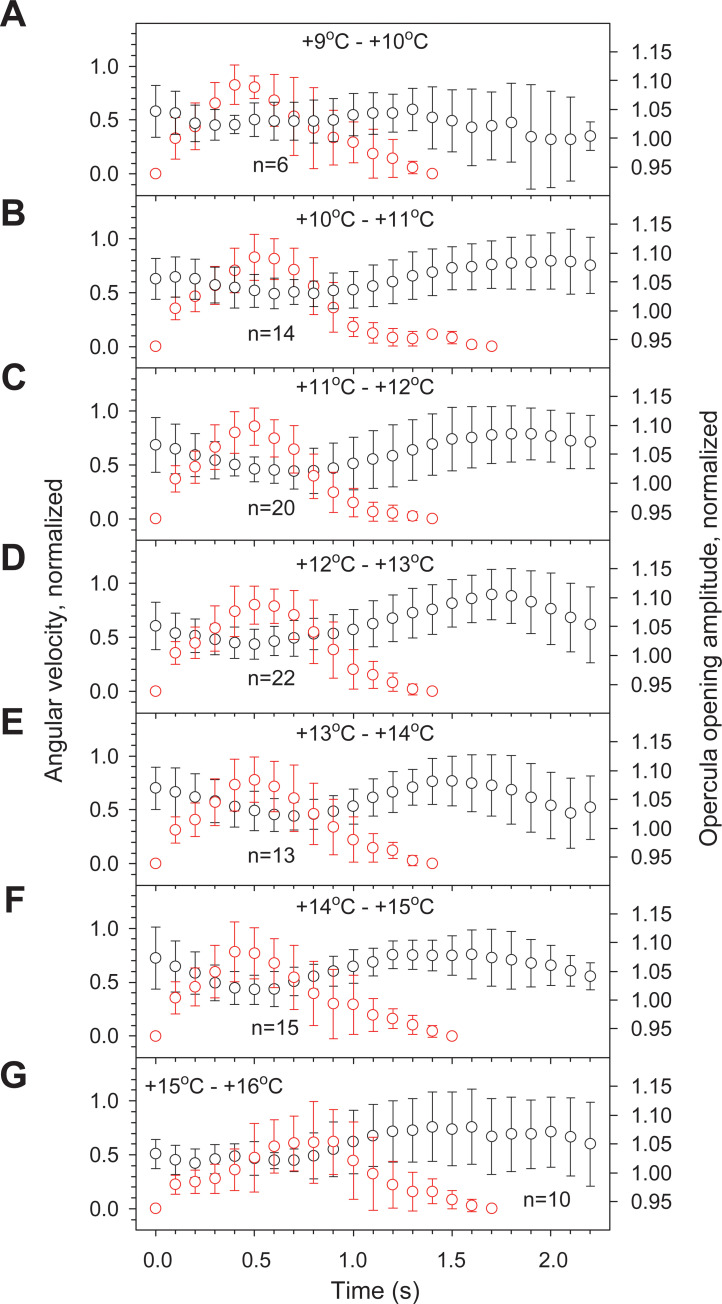
Respiratory-locomotor coupling in Hb+ *N*. *coriiceps* during thermally induced C-bend maneuvers. Data points and error bars represent mean and SD of angular velocity (red symbols) and opercula opening amplitude (black symbols), during C-bend turns of a single specimen of *N*. *coriiceps* (Cor #4 in S3 and S5 Figs depicting locomotor and respiratory responses), normalized to their maximum and minimum values, respectively, and averaged for all successive turns within each 1°C interval of temperature change (see numbers in the panels **(A)** through **(G)**). See S3 Table for numerical data.

In addition, this patterned behavior includes coordination between the movements of the opercula and rotation of the head. Namely, the acceleration and deceleration stages of head rotation during the C-bend turns are synchronized with the adduction and abduction phases of ventilatory opercular movements, respectively (Fig 8). That is, C-bend turns routinely commence with opercula partially open, though undergoing adduction and closing near the peak of angular velocity. Opercula start opening again during angular deceleration and continue to open after the turn is complete, achieving the maximally open state when the fish is gliding. Such synchronization is evident in most C-bend turns at temperatures between +9°C and +15°C (Fig 8A–8F), except within 1°C prior to LOE, when the synchrony is lost (Fig 8G). Characteristically, these triplet maneuvers synchronized with ventilatory movements of opercula involve only C-bend turns with V_a_ of ~250 deg·s^-1^, whereas V_a_ of S-bend turns (~1000 deg·s^-1^) is too fast to be coupled with opercula movements and gill ventilation.

## Discussion

Major findings of our study are two-fold: 1) Antarctic Notothenioid fishes respond to acute ambient warming with an extensive repertoire of maneuvers, most of which are observed in both Hb+ and Hb- species; 2) with some common tactics, behavioral strategies of Hb+ and Hb- fishes differ in terms of intensity, duration, thermal dependence and stereotypy of maneuvers recruited. These commonalities and differences are discussed below in relation to possible physiological and ecological correlates.

Locomotion has been long recognized as the most obvious, and probably most universal, behavioral response of fishes to environmental stressors, particularly temperature [48, 49]. From the rich repertoire of behaviors elicited in Hb- and Hb+ Antarctic fishes by acute warming, the earliest locomotor maneuvers in response to the initial temperature rise may represent the attempts at ecologically relevant avoidance reactions. These locomotor responses occur in all animals of both species with temperature elevations as little as 0.1°C, in agreement with early estimates of thermal sensitivity of fishes [48, 49]. Also called habitat selection, this behavioral response has been considered the most essential thermoregulatory mechanism of ectotherms in a heterogeneous thermal environment [13]. However, essential for meaningful interpretation of the results of this study in the context of thermal tolerance and vulnerability to climate change, we must emphasize that our acute experiments in the laboratory setting differ from ecologically relevant venues in several aspects. First, although allowed a certain degree of freedom to express various behaviors, the fishes in the experimental tank are prevented from performing thermoregulation by habitat selection and are forced to thermoconform. Second, warming continues during this obligatory thermoconformation, further exacerbating the environmental stress. Third, the rate of temperature increase (~3°C per hour) is at least 10^5^ times faster than any current estimate of the rate of the Southern Ocean warming due to climate change (~3°C per 100 years, [5–7]). Taking into account these aspects, we infer that most of the maneuvers of the fishes observed in our experiments are aimed to physiologically mitigate detrimental effects of unavoidable acute warming.

We reason that reduced motility following initial presumed avoidance reaction may represent a strategy for conserving energy and preventing metabolic stress, similar to transitory “quiescent behavior” observed in other fishes coping with progressive aquatic hypoxia [50, 51]. More prominent manifestation of this behavior in Hb- fishes may suggest that lower oxygen-carrying capacity of their blood makes them more “prudent”.

With regard to startle behaviors, they are generally considered in a relatively narrow context of predator-prey interactions [39] or in relation to direct external stimuli [40]. Hence, environmental factors, such as temperature or DO_2_, are usually viewed in terms of their adverse effects on the success in avoiding predation [52–54]. Thermally induced startle-like maneuvers in Antarctic fishes reported here for the first time suggest that similar behavioral patterns may be recruited not only in predator-prey interactions, but also in responses to environmental stress. Noteworthy in this regard, elevated temperature was reported to trigger escape-like turns in the African clawed frog tadpoles [55]. Nonetheless, since the fish in our experiments are not startled by an obvious stimulus, and their movements during these responses are relatively slow, we reason that these thermally-induced maneuvers are unlikely to represent *bona fide* startle behaviors (for recent definitions, see [56]).

It is indeed possible that startle-like behaviors may result from the direct effects of elevated temperature on peripheral and/or central components of underlying neural circuits, activation of sensorimotor code and/or related motor pattern generating systems. Notably, all Notothenioids examined to date appear to lack [57] the well identifiable T-shaped giant reticulospinal neurons in the hindbrain, the Mauthner cells, thought to initiate escape responses in most fishes [58, 59]. Absence of obvious Mauthner cells, or presence of cells with deviant anatomy, has been reported in some fishes [58, 59], and their escapes were slower and significantly delayed [60]. Our experiments do not provide any information about the latency of thermally induced startle-like maneuvers since they appear to be spontaneous rather than evoked by an obvious external stimulus other than temperature. Notably, absolute values of maximal linear velocity (0.7–1.4 m·s^-1^) attained by *N*. *coriiceps* during thermally induced S-bend startle-like maneuvers at +10 - +12°C are comparable with those during C-start escapes elicited by visual or tactile stimuli at 0°C in *N*. *neglecta* (1.28 m·s^-1^, [61]) and in *N*. *coriiceps* (0.71 m·s^-1^, [62]). Thus, at least during escape-like maneuvers, Hb+ Antarctic fishes appear to be capable of maintaining high motor performance within a range of acutely elevated temperatures, well above their natural limits. No data on kinematics of startle behaviors of Hb- icefish are available for comparison.

The significance of lateralization of startle-like behaviors in *N*. *coriiceps* and its reversal is not immediately apparent. Preferred direction away from the startling stimulus has been seen in other fishes [41]. Lateralization of barrier detours in consequent T-maze trials, on the other hand, appears to be poorly reproducible [63]. Neither phenomenon, however, appears to have a relation to lateralization of spontaneous thermally induced turns of *N*. *coriiceps* observed in our experiments. Otherwise, aquatic hypoxia as well as elevated temperature have been demonstrated to alter both the direction and magnitude of behavioral laterality [64, 65], considered mainly in the context of predator-prey relationships. While exact mechanisms leading to these alterations are yet to be established, behavioral laterality is thought to reflect asymmetrical functional specialization of the brain across vertebrate species [66], with a rightward bias being controlled by the left hemisphere which is thought to govern routine behaviors, and a leftward bias controlled by the right hemisphere presumably responsible for emergency and stress reactions [67].

Our findings of ventilatory adjustments while coping with progressive aquatic hypoxia and escalating metabolic load associated with rising temperatures are consistent with earlier observations in acutely warmed temperate (*e*.*g*., [68, 69]) and some Hb+ Antarctic fishes [70, 71]. Furthermore, Jayasundara et al. [71] report a bell-shaped thermal dependence of ventilation rate in another Hb+ Antarctic Notothenioid, *Trematomus bernacchii*, with a maximum near +8°C, comparable to our observations in *N*. *coriiceps*. Fast and substantial fall in *f*_*V*_ observed after the ventilation maximum in Hb+ *N*. *coriiceps* (but not in Hb- *C*. *aceratus*) may result, at least in part, from autonomic splenic contraction which can rapidly boost the blood oxygen carrying capacity, similar to that seen following acute step-wise transfer of *Trematomus bernacchii* to +10°C [72]. Noteworthy at this juncture, increased hematocrit has been reported in acutely warmed *N*. *coriiceps* at T_LOE_ [18, 73], although neither thermal dependence nor detailed physiology of the phenomenon are known. Otherwise, continued increase of ventilatory stroke volume (as deduced from measurements of OA, as a proxy metric) while maintaining nearly constant branchial pump suction (as deemed from measurements of OT, as an inverse proxy metric), evident in both Hb+ and Hb- species, suggests that during acute heat stress fishes employ additional, possibly more energetically advantageous, adjustments of ventilation by enhancing its efficacy (evident from changes in OV, as a proxy metric) without increasing *f*_*V*_. In addition, *N*. *coriiceps* may use synchronization of respiratory movements of opercula with head rotation during numerous C-bend turns to facilitate irrigation of the gills and thus increase respiratory efficiency via respiratory-locomotor coupling, comparable to that shown during undulatory swimming of a trout [74].

As for fanning, earlier observations of this behavior were made during egg-guarding, attributing it to parental care [46], with ventilation of spawn being considered the main purpose under conditions of hypoxia and CO_2_ build-up in the nest [75, 76]. Apparently widespread among Antarctic Notothenioids, egg-guarding does manifest uniparental fanning in some species [77, 78]. In our experiments, however, fanning occurs in all specimens, in the absence of clutch, *i*.*e*., under conditions that do not assume parental care. Based on the onset of fanning, nearly coincident with the maximum of *f*_*V*_ and greatly intensifying during subsequent hyperventilation plateaus, we hypothesize that it constitutes an alternative respiratory behavior in coping with thermal stress and progressive aquatic hypoxia. Supporting this hypothesis, continuous pectoral fanning was reported in captive *Trematomus loennbergii* in the McMurdo station aquarium [79], also without clutch. Although the significance of this behavior remained uncertain in this earlier report, it is plausible that some degree of hypoxia could exist in aquaria, even at low ambient temperature (*e*.*g*., due to overcrowding). Specific roles of fanning in respiration of Hb- and Hb+ fishes, however, may differ. High frequency undulatory fanning in *C*. *aceratus* may facilitate cutaneous gas exchange, the role of which has long been discussed, particularly in the physiology of scaleless Hb- channichthyids [17, 79]. Frequency of fanning in *N*. *coriiceps*, in contrast, is comparable with that of opercular beats, which may imply that in this species pectoral fin fanning may assist branchial pump.

Otherwise, all of these changes in respiratory behaviors of fishes observed in our experiments following the maximum of *f*_*V*_ between +8 and +8.5°C (*i*.*e*., after achieving the limit of ventilation effort) may be considered the manifestation of transition into “*pejus”* (from latin “worse”, [80]) range. Albeit during this range they appear to mitigate, at least in part, the deleterious effects of progressive aquatic hypoxia and escalating metabolic load, respiratory collapse inevitably occurs in both species when all adjustments fail precipitously prior to LOE. It is not clear, however, if this collapse results from the limitations in cardiac function experienced by the fishes or is, conversely, their cause.

Regarding fin splays, the ethology of this newly described behavior is not immediately apparent. We hypothesize that they may represent a possible contribution to cardiac output optimization during thermal stress, as they correspond to near maximal heart rates during thermal ramps [20]. Specifically, the extension of appendages may move pectoral muscles and thus expand the pericardium which is, in fishes, attached to muscular elements of the pectoral girdle. In effect, the dimensions of pericardia of fishes are finite, thus imposing a limit on the maximal cardiac stroke volume. Indeed, a general observation is that fishes respond to warming by increasing their heart rate, whereas the stroke volume appears to be thermally insensitive [11]. Some actively swimming fishes, including Hb- *C*. *aceratus*, however, have demonstrated distinct increases in stroke volume in response to acute warming, particularly near the peaks of their heart rates [20, 81–83]. Furthermore, surgical opening of the pericardium of a contracting *in situ* heart of Hb- *Channichthys rhinoceratus*, resulted in a collapse of the ventricle [84], demonstrating the involvement of the intrapericardial pressure in the filling of the heart chambers in this species. On the other hand, in *N*. *coriiceps*, splays may work alongside respiratory-locomotor coupling during repetitive startle-like maneuvers in Splay-Turn-Glide triplet FAP sequences, which persist in all Hb+ animals between ~12°C and ~15°C (over about one hour of warming ramp). On the other hand, while the sequence of the three components does not change with increasing temperature, the frequency of triplet occurrence and the duration of individual components within triplets vary, which is consistent with the concept of relative variability of FAPs [85].

These newly described maneuvers appear to involve multiple muscle groups (trunk, opercula, fins and, plausibly, heart) and thus may constitute more complex coordinated cardiac and respiratory mitigation of detrimental effects of acute heat stress. Notably, these FAP episodes disappear about 1° C prior to the onset of LOE, which may be indicative of a limitation in muscle performance at this temperature. With that said, isometric tension (*i*.*e*., force) produced *in vitro* by isolated single muscle fibers was shown to be maximal in a variety fishes at temperatures around those that occur in their natural habitat, but smaller at lower and higher temperatures [86–88], illustrating the stenothermal nature of muscle mechanics in both tropical and Antarctic fish. Maximum velocity of unloaded isotonic contraction, on the other hand, do correlate with temperature, showing no thermal compensation irrespective of species [87, 89–91]. Importance and complexity of the issue was accentuated in a recent review [92]. It has been, however, well recognized [93] that *in vitro* measurements of isometric and isotonic contraction in isolated fibers do not necessarily reflect how the muscles might function in the living animal, where a combination of both processes are seen in any cycle of contraction, especially in the muscle of swimming fish. With all that, further understanding of the effects of temperature on fish muscle mechanics and how it may requires additional investigation.

To summarize, we report that both Hb+ and Hb- Antarctic fishes respond to acute ambient warming with an extensive repertoire of behaviors. Restricted in their ability to perform essential thermoregulatory behavior such as habitat selection in a uniformly heated tank, fishes respond to progressive hyperthermia with an elaborate range of maneuvers—relative quiescence, startle-like behaviors, aquatic surface respiration, and pectoral fin fanning and splays, accompanied by continuous changes in ventilation. While manifestations of all these behaviors are species-specific in terms of intensity, duration, thermal dependence and stereotypy, most are observed in both fishes. To compensate for increasing respiratory demand at the temperatures above +8°C, both fishes enhance the efficacy of their respiratory pumps. In addition, sedentary Hb- fish supplement increased ventilatory effort with intense fanning which may facilitate cutaneous respiration, whereas more agile Hb+ fish augment it with respiratory-locomotor coupling during repetitive startle-like maneuvers. Taken together, the sum of these behaviors may be reasoned as metabolic, respiratory, cardiac, and hematologic (the latter only in Hb+ fish) accommodations, resulting in simultaneous concerted optimization of multiple vital functions, using species-specific behavioral strategies. In the face of continuous warming, however, the capacity of all these physiological adjustments is limited, and they only provide for short-term compensation in extreme conditions. Furthermore, with an apparent multitude of physiological functions involved, neither the cause of the ensuant organismal failure, nor the apparent differences in tolerance of acute sublethal thermal stress between the species can be attributed to a single organ or system.

Thus, our findings demonstrate considerable capacity of both Hb+ and Hb- Antarctic fishes for thermoconformation within a limited thermal range. While some of the temperatures achieved in our experiments are substantially higher than those the fishes are likely to encounter even in most dire scenarios of global warming, dramatic behavioral changes that we observe in both species are indicative of a variety of physiological adjustments triggered by warming over relatively brief periods. Moreover, these short-term behavioral and physiological adjustments may be imperative for transient migrations of eurybathic [22] Notothenioids to potential favorable niches within a changing thermo- and oxycline for efficient habitat selection. However, there remain many unknowns that may affect successful use of these new habitats under conditions of a lasting environmental change. In particular, while a potential to adapt to elevated temperatures has been suggested for some presumably highly stenothermal Antarctic species [71, 73, 94–102], the results of experimental studies underlying this conjecture (which, in fact, constitute another type of obligatory thermoconformation with more prolonged, but still miniscule on the ecologically relevant timescale, exposures to lesser warming) are somewhat uncertain and difficult to interpret, possibly due to differences in conditions and metrics used. Also, to date, there is no consensus about how the thermo- and oxycline may change in either persistent (*i*.*e*., gradual global climate change) or transitory (*i*.*e*., anomalous heat-wave like; [103, 104]) warming. It also remains be determined what particular challenges may arise in such a variety of scenarios, and which of the short-term adjustments described here the fish may (or may not) employ in each particular case. Our initial investigation does not answer directly any of these questions, but underscores the importance of additional bathymetric, physiological, ecological and behavioral studies on the subject matter.

Furthermore, it is reasonable to expect that adjustment of the fishes to putative new habitats should involve long-term behavioral and physiological adaptations, as well as ecological and evolutionary mechanisms [2, 21]. On the behavioral side, these adaptations may include adjustments of seasonal timing of life-history events (including reproduction) and biotic inter-species interactions (including predator-prey relationships). On the physiological side, development of long-term adaptations depends on the maintenance of successful, but usually rare, adaptive genetic variations [105], which is contingent on the large census and effective population sizes [106]. The latter may be particularly problematic for Antarctic and sub-Antarctic notothenioids, many of which remain depleted after severe industrial over-harvesting [107] in the 1960-80s, with yet unclear prospects for population recovery [108, 109]. At this juncture, we would like to underscore that, without rational science-based management of fisheries in the Southern Ocean [110, 111], unregulated anthropogenic interventions have the potential to produce irreversible damage to this ecosystem, irrespective of how well species can adapt to the detrimental effects of climate change.

## Supporting information

S1 Checklist (DOCX)

S1 Fig Metadata analyses of vertical temperature and DO_2_ profiles in areas of historic natural habitat of *N*. *coriiceps* and *C*. *aceratus*. **(A)** Vicinity of the Western Antarctic Peninsula; Data sources: February, 2002—Fritsen, C. (2011) CTD data from the Cruise CD-ROMS on ARSV Laurence M. Gould (LMG0201A, LMG0203, LMG0205, LMG0302, LMG0104) in the Southern Ocean from 2001–2003 (SOGLOBEC project). Biological and Chemical Oceanography Data Management Office (BCO-DMO). Dataset version 2011-04-07. Subset LMG0201 (Cast ## A1, A2, B2). http://lod.bco-dmo.org/id/dataset/2361; accessed 3/14/2017; July, 2001 and August, 2002—Klinck, J.M. and Hofmann, E.E. (2003) Processed standard depth (pressure) CTD data from SOGLOBEC survey cruises on RVIB Nathaniel B. Palmer—NBP0103; NBP0104; NBP0202; NBP0204—in the Southern Ocean from 2001–2002 (SOGLOBEC project). Biological and Chemical Oceanography Data Management Office. Dataset version 2003-06-13. Subsets NBP0104 and NBP0204. http://lod.bco-dmo.org/id/dataset/2360; accessed 3/17/2017. Data on pack ice situation in August 2002—U.S. Southern Ocean GLOBEC Report No. 8. Report of RVIB Nathaniel B. Palmer Cruise NBP02-04 to the Western Antarctic Peninsula, 31 July to 18 September 2002. http://www.ccpo.odu.edu/Research/globec/main_cruises02/nbp0204/menu.html. **(B)** Vicinity of the South Georgia Island; Data source—British Oceanographic Data Center, Natural Environment Research Council, UK. April, 1995, January, 2013 and March, 2015 and January, 2017 curves represent averages of STD/CTD data collected during the RRS James Clark Ross cruise JR19950320 (JR10) within The UK World Ocean Circulation Experiment (WOCE) Project (BODC ID 1011019, 1011020, 1011032, 1011044 and1011068); during RRS James Clark Ross cruise JR20130109 (JR274) within UK Ocean Acidification Research programme (BODC ID 1147675, 1147687, 1147699 and 1147706); during RRS James Clark Ross cruise JR20150309 (JR272D, JR310) within British Antarctic Survey Long Term Monitoring and Survey programme (BODC ID 1814444, 1814456, 1814468, 1814481 and 1814493); and during RRS James Clark Ross cruise JR16004 within the Ocean Regulation of Climate by Heat and Carbon Sequestration and Transports (ORCHESTRA) project of the Natural Environment Research Council, UK (BODC ID 1836882, 1836894, 1836901 and 1836925), respectively. January 2017 dataset does not have usable DO_2_ data. **(C)** Vicinity of the Bouvetøya Island; Data source—British Oceanographic Data Center, Natural Environment Research Council, UK. Curves represent averages of STD/CTD data collected during the Marion Dufresne cruise MD166 (BONUS-GOODHOPE, GIPY04) within the GEOTRACES project of the University of Western Brittany, France (BODC ID 1105470, 1105494, 1105433). Temperature of surface waters varies between locations during austral summer. Surface water DO_2_ in the vicinity of the Western Antarctic Peninsula does not vary much between seasons, but depends on pack ice cover (compare July, 2001 and August, 2002 DO_2_ profiles in a, right panel). No STD/CTD data are available for austral winter in the vicinity of South Georgia and Bouvetøya islands. Preferred bathymetric ranges of *Nototheniidae* and *Channichthyidae* (<200 meters for *N*. *coriiceps* and >100–200 meters for *C*. *aceratus*; Hureau, 1985), correspond to pronounced thermo- and oxycline in all locations.
(EPS)

S2 Fig Detailed view of experimental tank. **(A)** Overview of the tank and video registration system. **(B)** Heat exchanger with recirculation system.
(TIF)

S3 Fig Locomotor responses to warming in different specimens of Hb+ *N*. *coriiceps* and Hb- *C*. *aceratus*. Traces are temperature plots of instantaneous (30 Hz sampling rate) velocity in individual experiments with **(A)** five specimens of N. coriiceps and **(B)** five specimens of C. aceratus, normalized for body length (BL) of the specimen.
(TIF)

S4 Fig Progressive aquatic hypoxia concomitant with warming of water in the experimental tank. Solid black lines and grey error bars in the graphs represent means and SEM of absolute DO_2_ measured simultaneously with temperature ramps, averaged between five experiments of each species. Dashed lines represent theoretical temperature plots of DO_2_ calculated using Henry’s law, corrected for salinity. For conventions of analyses and presentation of grouped data as a function of temperature, Materials and Methods.
(EPS)

S5 Fig Ventilatory responses to warming in different specimens of Hb+ *N*. *coriiceps* and Hb- *C*. *aceratus*. Data points represent ventilation rates (f_V_), amplitudes of opercula opening (OA), and opening time of opercula (OT) in three specimens of each species (## in legends correspond to individual experiments shown in S3 Fig).
(EPS)

S6 Fig Aquatic surface respiration of Hb+ N. coriiceps and Hb- C. aceratus as a function of temperature. Data points represent time spent by three specimens of each species (labelled corresponding to S3 Fig) at water-air interface in a form of continuous uninterrupted surfacing bouts.
(EPS)

S1 Movie Fanning of Hb- *C*. *aceratus* at +10.5°C–side view. (MOV)

S2 Movie Fanning of Hb- *C*. *aceratus* at +10.5°C–ventral view. (MOV)

S3 Movie Fanning of Hb+ *N*. *coriiceps* at +11.2°C. (MOV)

S4 Movie *S*plays and FAPs of Hb+ *N*. *coriiceps* at +14.7°C. (MOV)

S1 Table Numerical data for T_LOE_ values in individual experiments with Hb+ *N*. *coriiceps* and Hb- *C*. *aceratus*. (XLSX)

S2 Table Numerical data for respiratory metrics in individual experiments with Hb+ *N*. *coriiceps* and Hb- *C*. *aceratus*. (XLSX)

S3 Table Numerical data for respiratory metrics (HWnorm) and angular velocities (Vnorm) during thermally induced C-bend maneuvers of a single specimen of Hb+ *N*. *coriiceps*. (XLSX)
