# Supplementary figures and images for: Antarctic teleosts with and without hemoglobin behaviorally mitigate deleterious effects of acute environmental warming
Source: PLoS One. 2021 Nov 24;16(11):e0252359. doi: 10.1371/journal.pone.0252359 (PMC8612528; doi:10.1371/journal.pone.0252359)

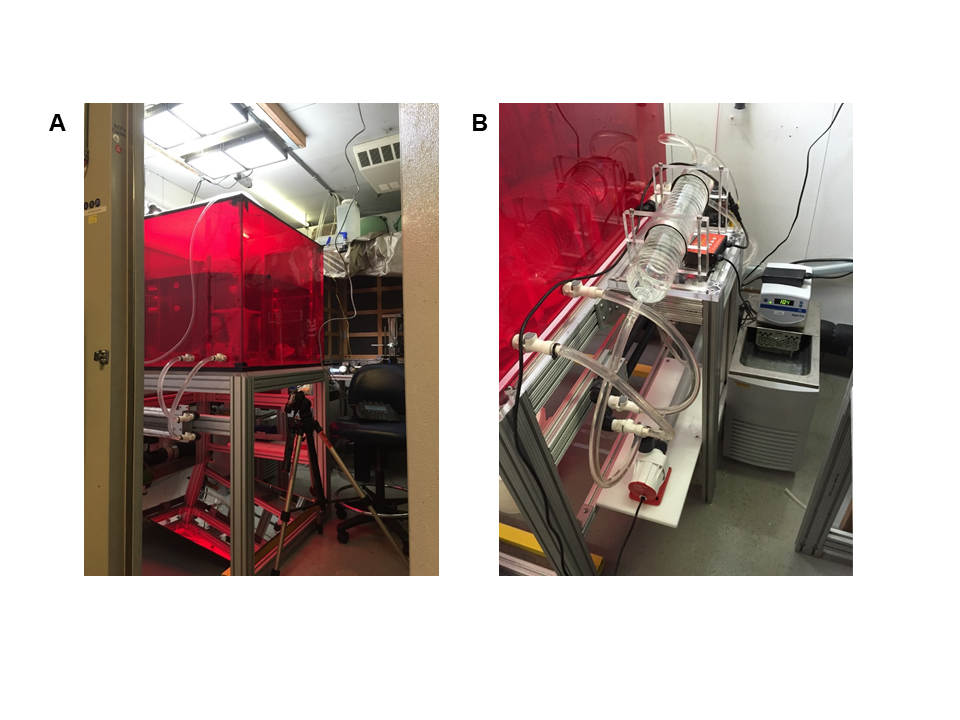

Supplement: S2 Fig — (A) Overview of the tank and video registration system. (B) Heat exchanger with recirculation system. (TIF) [file pone.0252359.s003.tif]

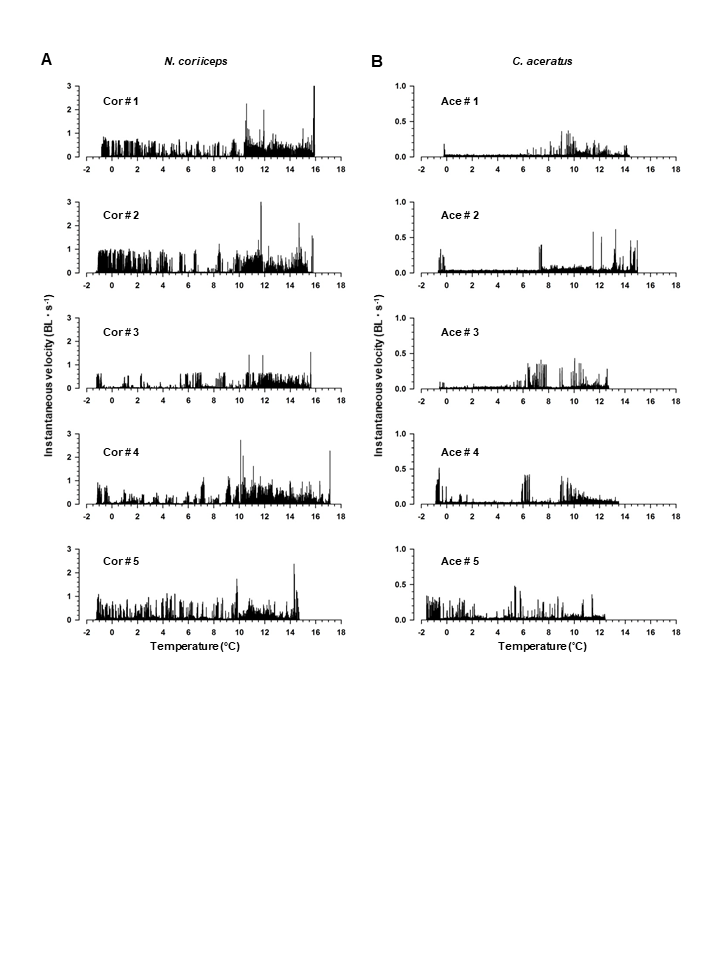

Supplement: S3 Fig — Traces are temperature plots of instantaneous (30 Hz sampling rate) velocity in individual experiments with (A) five specimens of N. coriiceps and (B) five specimens of C. aceratus, normalized for body length (BL) of the specimen. (TIF) [file pone.0252359.s004.tif]
